# Measuring antibody avidity to *Plasmodium falciparum* merozoite antigens using a multiplex immunoassay approach

**DOI:** 10.1186/s12936-020-03243-3

**Published:** 2020-05-01

**Authors:** Diane Wallace Taylor, Naveen Bobbili, Alex Kayatani, Samuel Tassi Yunga, Winifrida Kidima, Rose F. G. Leke

**Affiliations:** 1 Department of Tropical Medicine, Medical Microbiology, and Pharmacology, John A. Burns School of Medicine, University of Hawaii, 561 Ilalo Street, Honolulu, HI 96813 USA; 2 Faculty of Medicine and Biomedical Sciences, The Biotechnology Center, University of Yaoundé 1, Yaoundé, Cameroon

**Keywords:** Malaria, *Plasmodium falciparum*, Antibodies, Antibody avidity, Multiplex immunoassay, Methods study

## Abstract

**Background:**

Antibodies (Ab) play a significant role in immunity to *Plasmodium falciparum* malaria. Usually, following repeated exposure to pathogens, affinity maturation and clonal selection take place, resulting in increased antibody avidity. However, some studies suggest affinity maturation may not occur to malaria antigens in endemic areas. Information on development of antibody avidity is confusing and conflicting, in part, because different techniques have been used to measure avidity. Today, bead-based multiplex immunoassays (MIA) are routinely used to simultaneously quantitate antibody levels to multiple antigens. This study evaluated the feasibility of developing an avidity MIA with 5 merozoite antigens (AMA1, EBA-175, MSP1-42, MSP2, MSP3) that uses a single chaotropic concentration.

**Methods:**

The most common ELISA protocols that used the chaotropic reagents guanidine HCl (GdHCl), urea, and ammonium thiocyanate (NH_4_SCN) were adapted to a multiplex MIA format. Then, different concentrations of chaotropes and incubation times were compared and results were expressed as an Avidity Index (AI), i.e., percentage of antibody remaining bound in the presence of chaotrope. Experiments were conducted to (i) identify the assay with the widest range of AI (discriminatory power), (ii) determine the amount of chaotrope needed to release 50% of bound Ab using plasma from adults and infants, and (iii) evaluate assay repeatability.

**Results:**

Overall, 4 M GdHCl and 8 M urea were weaker chaotropes than 3 M NH_4_SCN. For example, they failed to release significant amounts of Ab bound to MSP1-42 in adult plasma samples; whereas, a range of AI values was obtained with NH_4_SCN. Titration of NH_4_SCN revealed that 2 M NH_4_SCN gave the widest range of AI for the 5 antigens. Binding studies using plasma from 40 adults and 57 1-year old infants in Cameroon showed that 2.1 M ± 0.32 (mean ± SD) NH_4_SCN (adults) and 1.8 M ± 0.23 M (infants) released 50% of bound Ab from the merozoite antigens.

**Conclusions:**

An avidity MIA is feasible for the 5 merozoite antigens that uses a single concentration (2 M) of NH_4_SCN. The assay provides a simple method to quickly obtain information about Ab quantity and quality in the acquisition of immunity to malaria in endemic populations.

## Background

Antibodies (Ab) play a critical role in immunity to *Plasmodium falciparum*, primarily by blocking key epitopes on merozoites, preventing cytoadherence of infected erythrocytes, and enhancing phagocytosis. Antibody avidity (or functional affinity) is the net antigen-binding force of populations of Ab in sera [1]. Thus, studying antibody avidity provides insight into the extent of somatic mutation of immunoglobulin hypervariable regions and subsequent clonal selection. Considering the importance of Ab in immunity to malaria, including to merozoite antigens, surprisingly little is known about antibody avidity in naturally-infected individuals. Early studies showed that Ab avidity to an extract of *P. falciparum*-infected erythrocytes increased after a few infections in a low transmission region [2, 3]. However, in high transmission areas, most studies have found little or no increase in antibody avidity with age to merozoite antigens, including MSP1, MSP2, MSP3 and EBA-175 [4–9], although an increase with age for AMA1 has been reported [4, 5]. Thus, there is much to be learned about the role of Ab avidity in immunity to malaria.

A variety of protocols have been used to study antibody avidity. Although a few investigators have employed plasma magnetic resonance or bilayer interferometry [4, 7, 10], most studies have used the ELISA format with different concentrations of chaotropic agents [2, 3, 5, 6, 8, 9, 11–17]. In these assays, results are often expressed as an Avidity Index (AI), defined as the amount (e.g., O.D.) of Ab remaining bound to an antigen in the presence of a chaotropic agent divided by amount of Ab bound in its absence multiplied by 100. Studies of avidity to merozoite antigens with plasma from naturally-infected humans have used a variety of chaotropes, including 0.5 M, 2 M, 4 M, or 5 M guanidine HCl (GdHCl) [5, 6, 9, 11]; 8 M urea [2, 14–17]; or 1 M or 2.4 M thiocyanate (SCN) [3, 8]. Additional studies of other malarial antigens have used 3 M NH_4_SCN for VAR2CSA [18, 19] and 1 M NH_4_SCN for volunteers immunized with the RTS/S vaccine [12, 13]. A few studies, mainly laboratory-based, employed titration curves of urea or NH_4_SCN to determine amount of chaotrope needed to release 50% of bound Ab, usually in animal models vaccinated with malaria antigens [20, 21]. Prior avidity studies have usually considered only 1 to 2 antigens, have not explained why the chaotrope was selected, or have compared results among various chaotropes for the same or multiple antigens. The use of different methodologies makes it impossible to compare results between studies.

Today, bead-based multiplex immunoassays (MIA) are commonly used to measure Ab to combinations of malarial antigens [22–26]. Currently, it is unclear if an avidity MIA with multiple malaria antigens can be developed that uses only one concentration of a chaotropic agent, since prior avidity studies of viral and bacterial antigens have found that different concentrations of salt were required to release 50% of bound Ab from different antigens [1, 27].

This study explored the feasibility of an avidity MIA for 5 *P. falciparum* merozoite proteins (AMA1, EBA-175, MSP1-42, MSP2 and MSP3). The goal was to determine if accurate information could be obtained using a single concentration of only a single chaotrope. First, the most commonly used ELISA protocols for GdHCl, urea, and thiocyanate were adapted to the MIA format. These results allowed comparison of the amount of Ab released by the different chaotropes. Next, titration curves for GdHCl, urea and NH_4_SCN were examined; as well as, the length of incubation with different salt concentrations. Initial results revealed that 2 M NH_4_SCN consistently gave the widest range of AI values. Subsequent experiments determined (i) how 2 M NH_4_SCN compared with the amount of NH_4_SCN required to release 50% of Ab bound to the antigens, (ii) if the assay was appropriate for measuring avidity in infants with newly-developing malarial immunity as well as adults with high levels of immunity, and (iii) the reproducibility of the avidity MIA. Overall, the combined results lead to the conclusion that 2 M NH_4_SCN provides meaningful results in an avidity MIA for the 5 *P. falciparum* merozoite recombinant proteins evaluated.

## Methods

### Multiplex immunoassay

Details of the basic MIA using *P. falciparum* recombinant proteins was originally described in 2006 [22]. Characteristics of the recombinant proteins have also been detailed previously [22, 28] and include: AMA-1 (3D7), EBA-175, MSP1-42 (3D7), MSP2 (FcR3), and MSP3 (HB3). In brief, SeroMap beads (Luminex) with different spectral addresses were covalently coupled with saturating (i.e., optimal) amounts of recombinant proteins and pooled to create a 5-plex containing 2000 beads of each antigen in 50 µl. As reported previously, the 5-plex was compared with individual antigens (mono-plex) to ensure competition among antigens did not occur [22].

The antibody levels were determined by combining 50 µl of plasma (diluted 1:300 with PBS plus 1% BSA, pH 7.2) with 50 µl of the 5-plex bead mixture (total plasma dilution of 1:600) in filter microtitre plates. Following incubation at room temperature on a rotary shaker for 1 h, beads were washed twice with PBS + 0.05% Tween 20 and once with 1%BSA in PBS. Then, 100 µl of secondary antibody (R-phycoerythrin-conjugated, Affini Pure F(abʹ)_2_ fragment, Goat anti-human IgG Fc fragment specific, Jackson Immunoresearch, West Grove, PA) diluted to 2 µg/ml in PBS-1% BSA was added to each well and incubated in the dark on a rotary shaker for 1 h. Beads were washed, re-suspended in 100 µl PBS-1% BSA, and 85 µl of the microsphere suspension was analyzed using a Liquichip M100 reader (Qiagen, Valencia, CA). The reader was programmed to read a minimum of 100 beads per spectral address, DD Gate 7500–15,000. Results were expressed as median fluorescence intensity (MFI). The linear part of the Ab binding curve (MFI) was determined for each antigen and found to extend from ~ 1000 to > 20,000 MFI with r coefficients ranging between 0.913 and 0.995 (Additional file 1: Fig. S1).

### Basic avidity MIA

The avidity MIA assay was performed as described above, with the inclusion of an additional step. After incubation with plasma, the bead-Ab complexes were washed and then incubated for 30 min with 100 µl of PBS (no salt) or specified concentrations of different chaotropes (with salt). After washing, 100 µl of R-phycoerythrin-conjugated goat-anti-human IgG (Fcγ specific) was added to beads for 1 h incubation. Percentage of high avidity Ab was calculated by the formula (AI = [MFI beads with salt]/[MFI beads with no salt] × 100). Since AI evaluate strength of Ab binding and MFI indicate the amount of Ab, the two measures should be independent. Thus, in investigating the feasibility of an avidity MIA, it was important to confirm independence of MFI and AI. Results showed that similar AI were obtained when different dilutions of the same plasma sample was used (Additional file 2: Fig. S2).

### Plasma samples

A collection of de-identified, archival, plasma samples from malaria endemic regions of Cameroon with high transmission were used [29, 30]. Samples included: plasma from 5 selected adults with known Ab levels for the antigens; 40 adults residing in Ngali II (pregnant women, non-pregnant women, males) who had been repeatedly infected with malaria and had developed high malarial immunity, and 57 12-month-old infants living in Ngali II who had just begun developing immunity to malaria [30]. Two pools of plasma were used as positive controls (PC), with PC consisting of plasma samples pooled from adults living in Ngali II village (a high malaria transmission area), Cameroon and positive control #2 (PC#2) including plasma pooled from pregnant women living in the same village [29]. Negative controls (NC), used to establish a cut-off for positivity, included plasma samples from US individuals who had never been exposed to malaria.

### Developing the avidity MIA

**Fig. 1:**
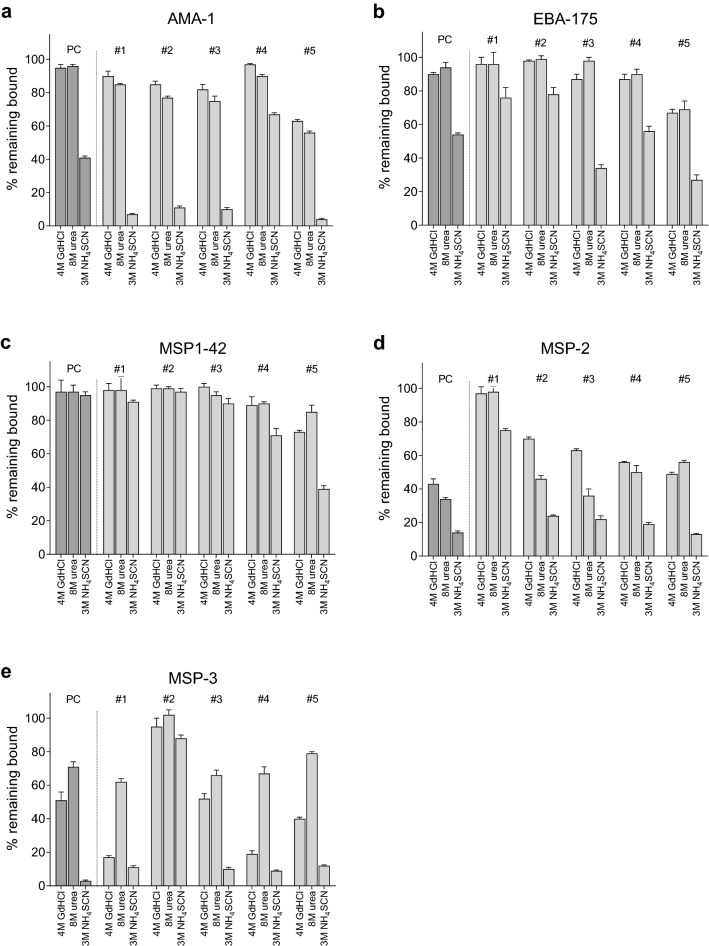
Comparison of avidity indexes for 5 merozoite antigens using 3 different chaotropic agents. Antigen-coupled beads were incubated with a 1:600 dilution of the positive control (PC) and plasma from adults (#1–#5) living in a high malaria transmission area in Cameroon. After incubation, Ab-bound beads were incubated with 4 M GdHCl for 10 min, 8 M urea for 10 min, or 3 M NH_4_SCN for 30 min. Results show mean AI values (i.e., the percentage of Ab remaining bound after treatment with salt) ± SD for 3 replicate samples. Antigens included: AMA1 (3D7), EBA-175, MSP1 (3D7), MSP2 (FC27) and MSP3 (HB3). Baseline (no salt) MFI values [mean ± SD] for the 6 plasma samples (PC + 5 adults) were as follows: **a** AMA1 = 23,677 ± 1079 MFI; **b** EBA-175 = 22,002 ± 1573 MFI; **c** MSP1 = 11,196 ± 1405 MFI; **d** MSP2 = 5702 ± 2282; and **e** MSP3 = 4128 ± 1232 MFI

**Fig. 2:**
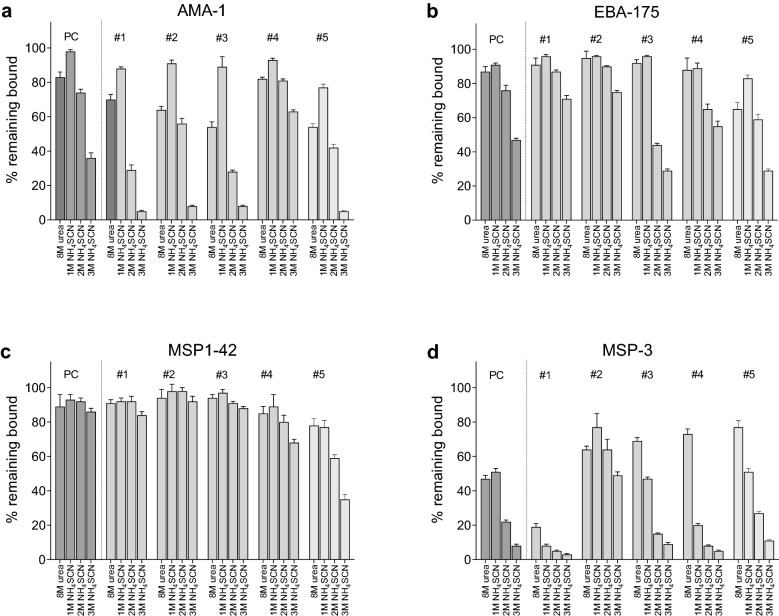
Avidity indexes after treatment with 8 M urea and 1 M, 2 M and 3 M NH_4_SCN. The same PC and samples from 5 individual adults used in Fig. 1 were re-tested in this experiment. Following incubation of plasma with the antigen-coupled beads, the complexes were incubated with 8 M urea or 1 M, 2 M and 3 M or NH_4_SCN for 30 min and amount of Ab remaining bound (AI) were calculated. Error bars represent the mean ± one SD for 3 replicates. Baseline MFI values [mean ± SD based on 15 replicates in the absence of salt] were as follows: **a** AMA-1 23,958 ± 98 MFI; **b** EBA-175: 22,413 ± 166; **c** MSP1 3D7: 10,169 ± 208 MFI; **d** MSP3: 2122 ± 82MFI. OPTIONAL INSERT: Comparison of data in Figs. 1 and 2 show that the majority of mean AI have overlapping SD; however, a few differences are seen, especially with 8 M urea for AMA1 and MSP3 that are due to inter-assay variation

**Fig. 3:**
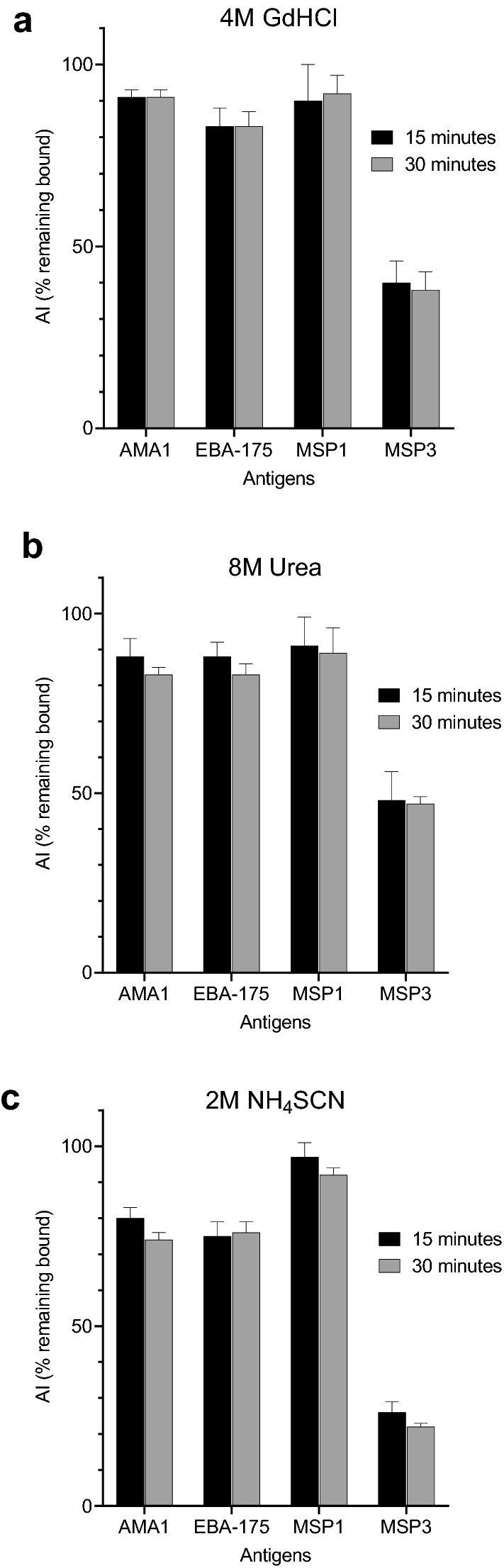
Comparison of the length of incubation with the chaotrope. Antigen-coupled beads were incubated with the pooled Positive Control (PC) for 60 min. Beads were then washed, incubated with 4 M GdHCl, 8 M urea, or 1 M, 2 M or 3 M NH_4_SCN for either 15 or 30 min at RT on a rotary shaker. Following washing and incubation with R-phycoerythrin-labeled, Goat anti-human IgG (Fc fragment) for 60 min, MFI were recorded and AI calculated. Error bars represent mean ± SD based on 3 replicates. Baseline MFI values [mean ± SD MFI for 30 replicates in the absence of salt] were as follows: AMA-1 23,955 ± 133 MFI; EBA-175 22,473 ± 587; MSP1 (3D7) 10,042 ± 333 MFI; and MSP3: 1990 ± 155 MFI

**Fig. 4:**
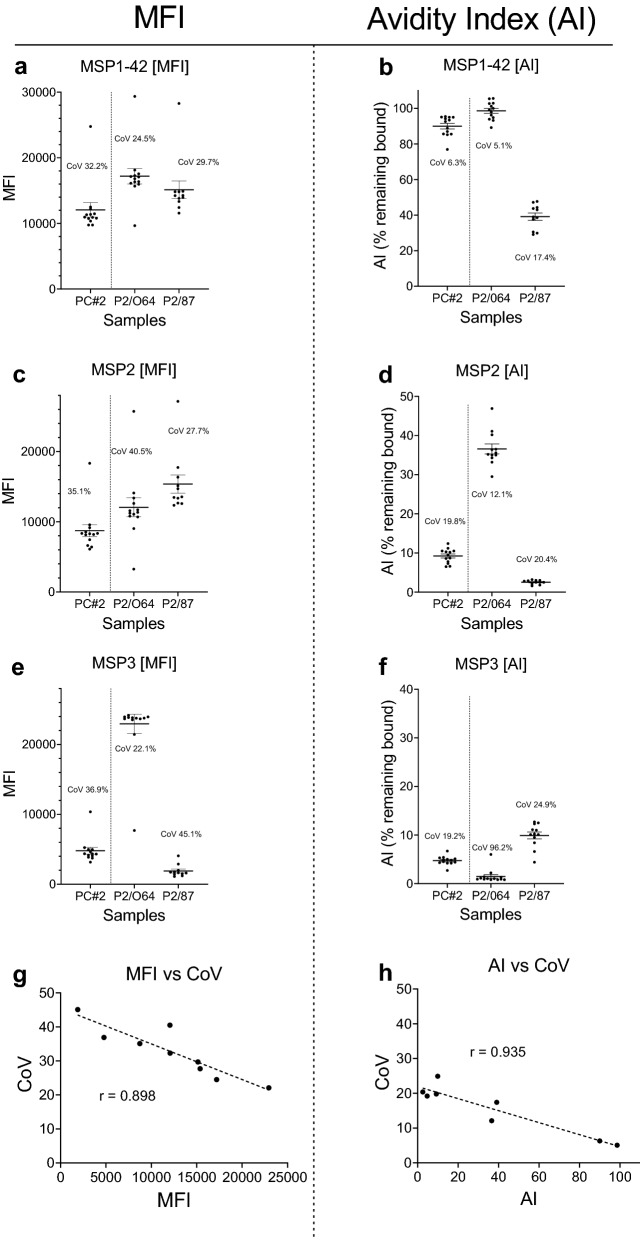
Variation among replicate assays. A second Positive-Control (PC#2) and plasma from two adults (P2/064, P2/87) were used as positive controls on 13 plates in a separate avidity study conducted over a 30-day period. Horizontal bars represent means and SD for 13 replicates. Bottom 2 figures are based on the 9 samples shown in the figures above; trendline represents a simple linear regression

The initial experiment adapted 3 ELISA protocols for measuring Ab avidity to the MIA format. The protocol employed 4 M GdHCl for 10 min [5, 11], 8 M urea for 5–10 min [2] and 3 M NH_4_SCN for 30 min [18, 19] (Fig. 1). In the second set of experiments, 8 M urea, and 1 M, 2 M and 3 M NH_4_SCN were compared using the following conditions: 50 µl of diluted plasma was incubated with 50 µl Ag-coupled bead mixture for 60 min; washed three times; beads were re-suspended in 100 µl of the chaotropic agent for 30 min; beads were washed and incubated with 100 µl of PE-anti-human IgG for 60 min; washed and examined using a MicroChip 100 as described above (Fig. 2). In the third experiment, 2 M, 4 M and 8 M GdHCl, 4 M, 8 M and 12 M urea, and 1.5 M, 3 M and 4.5 M NH_4_SCN were evaluated using the above protocol (Additional file 3: Fig. S3). In the fourth experiment, the above protocol was followed except beads were incubated for either 15 or 30 min with the respective chaotropic agents to evaluate the effect of time of exposure to various concentrations of chaotrope (Fig. 3). To determine the amount of NH_4_SCN needed to remove 50% of bound Ab from the merozoite antigens, titrations curves of 0 M, 1.5 M, 3 M and 4.5 M NH_4_SCN were constructed (as described in “Results” section) and the Forecast program was used to estimate the 50% concentration (results in Table 1). Finally, to evaluate the repeatability of the assay over time, archival data from 13 avidity assays, conducted over a 30-day period using MSP1-42, MSP2 and MSP3, were evaluated (Fig. 4).

**Table 1:** Molar amount of NH_4_SCN needed to release 50% of bound antibody

N = 40 adults	AMA1	EBA-175	MSP1-42	MSP2	MSP3
% Ab positive	100 (40/40)	90 (36/40)	100 (40/40)	98 (39/40)	68 (27/40)
Median MFI (95% CI)^a^ (1:1000 dilution)	16,799 (13,176, 18,855	13,815 (11,189, 17,637)	9054 (6907, 14,221)	9177 (5309, 11,151)	2066 (1287, 3477)
Molars of chaotrope to release 50% Ab bound (mean ± SD)	2.31 M ± 0.44	2.25 ± 0.33	2.32 ± 0.82	1.71 ± 0.22	1.70 ± 1.72
n = 57 1-year old infants	AMA1	EBA-175	MSP1-42	MSP2	MSP3
% Ab positive	72 (41/57)	49 (28/57)	9353/57	88 (50/57)	37 (21/57)
Median MFI (95% CI)^a^ (1:100 dilution)	9664 (5717, 13,906)	1094 (673, 1584)	8974 (4365, 11,808)	5536 (2335, 9276)	1247 (750, 1653)
Molars of chaotrope to release 50% Ab bound Ab	1.78 M ± 0.27	2.0 ± 0.50	2.1 ± 0.59	1.6 ± 1.1	1.6 ± 0.12

^a^95% confidence interval (CI) of the median based on Ab-positive samples

### Statistical analysis

Antibody values were recorded as median fluorescence intensity (MFI). The avidity Index (AI) was calculated as the MFI of the sample treated with chaotrope divided by the MFI in the absence of chaotrope times 100. Results from replicate assays were expressed as means and standard deviations (SD). Median MFI and AI values for adults and infants were reported (not normally distributed). The estimated amount of salt needed to remove 50% of bound Ab was determined using the Forecast Program (Excel).

## Results

### Comparison of the three different chaotropes

In the initial experiment, ELISA protocols for 4 M GdHCl, 8 M urea and 3 M NH_4_SCN were adapted to the MIA format (see “Methods” section for details). The avidity MIA included the malaria antigen 5-plex, the PC and 5 selected plasma samples with high Ab levels (Fig. 1). The PC was pooled from multiple adults and represents Ab in the “general population;” whereas, the 5 individual samples provided information about variation among different adults. Treatment of the PC with 4 M GdHCl and 8 M urea resulted in AI > 80 for AMA1, EBA-175 and MSP1-42 (i.e., > 80% of Ab remained bound); AI of 40 to 50 to MSP3; and 35 to 40 for MSP2 (Fig. 1). In contrast, treatment with 3 NH_4_SCN substantially reduced Ab binding with AI of 40 to AMA1, 50 to EBA-175, 15 to MSP2, and 15 to MSP3 (5), but AI > 90 remained to MSP1-42. Thus, NH_4_SCN was a stronger chaotrope than the other two reagents for AMA1, EBA-175, MSP2, and MSP3.

As expected, a pattern similar to that with PC was observed among the 5 individuals, but substantial variation was also found. The 5 adults had different proportions of high avidity Ab (i.e., AI) for each of the antigens. For example, after 3 NH_4_SCN treatment, person #1 had 92% high avidity Ab to MSP1-42, 87% to EBA-175, but only 5% to MSP3. Thus, it was possible for a person to have a high AI for one antigen and relatively fewer high avidity Ab to another antigen. Likewise, the AIs for MSP3 were generally lower than for the other antigens, except for individual #2 who had AIs over 80 for MSP3 (Fig. 1e). Thus overall, AI for some antigens were higher than for others. In summary, the PC reflected the effect of combining plasma samples with different proportion of high avidity Ab, i.e., it did not have the highest AIs, but rather the average of the combined samples. Additionally, significant variation in AIs was observed among individuals who had been exposed malaria all their lives.

Because of the relatively strong effect of 3 M NH_4_SCN compared to GdHCl and 8 M urea, a second experiment was conducted using the same plasma samples treated with 8 M urea and a titration of NH_4_SCN (1 M, 2 M and 3 M) (Fig. 2). In general, the effectiveness of 1 M NH_4_SCN and 8 M urea were similar for the 4 antigens (MSP1-42, EBA-175, MSP2 and AMA1), while the 3 concentrations of NH_4_SCN gave a nice titration, especially for AMA-1, EBA-175, and MSP-3, with most Ab remaining bound with 1 M NH_4_SCN, intermediate amounts at 2 M, and substantial amounts of Ab being removed with 3 M. Overall, 2 M NH_4_SCN provided the widest range of AIs (i.e., had the best discriminatory potential).

To further explore the influence of chaotrope concentration, one-half and twice the molar concentrations of 4 M GdHCl, 8 M urea and 3 M NH_4_SCN were evaluated on MSP1, MSP2 and MSP3 as test antigens (Additional file 3: Fig. S3). NH_4_SCN was the only chaotrope that dislodged Ab bound to MSP1, but at 3 M and 4.5 M released almost all Ab bound to MSP2 and MSP3. Urea remained the least stringent chaotrope, failing to release Ab from MSP1 at any concentration, and provided a weak dose–response with MSP2 and MSP3 at 8 M and 12 M (Additional file 3: Fig. S3). In contrast, 4 M and 8 M GdHCl appeared to release almost all Ab bound to MSP2 and MSP3, but only 10–20% of Ab bound to MSP1 (Additional file 3: Fig. S3). Overall, none of the concentrations of the 3 chaotropes improved the range of AI compared to 2 M NH_4_SCN (Fig. 2).

### Timing of incubation with chaotropes

Previous studies used various lengths of time for incubating antigen-Ab complexes with chaotropes, with some protocols simply washing bound antigen-Ab complexes with the salt solution whereas others incubated for 10, 15 or 30 min. The most common lengths were 10–15 and 30 min. In this experiment, Ab-antigen-bead complexes were incubated for 15 and 30 min with either 4 M GdHCl, 8 M urea or 2 M NH_4_SCN (Fig. 3). When directly compared in this experiment, results showed that AIs were only slightly, but not significantly, lower after 30 compared to 15 min of incubation regardless of the chaotrope. In subsequent experiments, a 30-min incubation period was used for convenience when conducting large multiple assays.

### The molar amount of NH_4_SCN needed to release 50% of bound antibody

In this experiment, 40 plasma samples from adults living in the rural village of Ngali II were screened in the avidity MIA using 3 concentrations of NH_4_SCN (1.5 M, 3.0 M and 4.5 M) and (i) Ab prevalence, (ii) levels and (iii) for Ab-positive samples the amount of salt needed to release 50% of bound Ab was calculated (Table 1). Overall, 90–100% of adults had Ab to AMA1, EBA-175, MSP1 and MSP2, but only 68% had Ab to MSP3. Similar median Ab levels (MFI) were detected to AMA1 and EBA-175 (16,799 and 13,815 MFI); equivalent median Ab levels to MSP1 and MSP2 (9054 and 9177); and lower levels of Ab to MSP3 (2066 MFI). The molar concentration estimated to obtain AI of 50 ranged from 1.7 to 2.3 M (Table 1), with a mean of 2.1 M ± 0.32 (mean ± SD) for adults living in a malaria-endemic area. Thus, 2 M NH_4_SCN appeared to release about half of the Ab bound to the 5 antigens, regardless of Ab levels.

The above plasma samples were from adults who had been exposed to *P. falciparum* throughout their lives and, therefore, most likely had acquired a mature malarial humoral response over time. The question then became, would 2 M NH_4_SCN be appropriate for infants who were just beginning to acquire immunity? Therefore, 57 plasma sample were screened from infants who were 1 year of age (mean ± SD: 362 ± 12 days; range: 326–385 days) living in Ngali II, who had been followed since birth and had begun to make their own Ab after the decline of maternal antibodies. As expected, the majority of infants had Ab to AMA1 (72%), MSP1-42 (93%) and MSP2 (88%); with fewer infants having Ab to EBA-175 (49%) and MSP3 (37%). Ab levels were much lower in the infants to all of the antigens compared to adults (Table 1), but the amount of NH_4_SCN needed to release 50% of bound Ab to the antigens was slightly lower, ranging from 1.6 to 2.1 M (compared to 1.7 to 2.3 in adults; Table 1), with a mean of 1.8 M ± 0.23 M. Although lower in young children, assay conditions to release 50% of bound Ab did not differ drastically between infants and adults.

### Reproducibility of the avidity MIA

In a separate study on Ab avidity, MSP1-42, MSP2 and MSP3 were included as antigens on 13 different plates run over a 30-day period. The protocol included incubating antigen-Ab for 60 min, then 30 min with 2 M NH_4_SCN, followed by 60 min with secondary Ab. The archival data provided information on day-to-day and plate-to-plate variation of the avidity MIA assay. Figure 4 shows the variation in MFI and AI (mean ± SD) for 13 replicate assays. Coefficient of Variation (CoV) for each of the AIs was smaller than the corresponding CoV for MFI, e.g., the MFI CoV for MSP1-42 with PC#2 was 32.2%; whereas, the AI CoV was 6.3% (Fig. 4a, b). A few MFI value were outside of the mean ± 1 SD, but the corresponding AI remained within, demonstrating the independence of the two measures. Direct linear relationships with higher MFI (r = 0.898) and AI (r = 0.936) values and smaller CoV were detected (Fig. 4g, h). Thus, repeating the avidity MIA using 2 M NH_4_SCN for 30 min resulted in similar avidity indexes.

## Discussion

Considering the importance of Ab in immunity to malaria and the extensive search for correlates for protection, it is surprising that Ab avidity has received relatively little attention. The exclusion method used in this study is a simple approach for measuring Ab avidity, whereby Ab-antigen complexes are treated with a chaotrope and the amount of Ab release is determined. Although the exact mechanism is unknown, it is hypothesized that the gap between antigens and bound Ab with good complementarity is tight enough to exclude the denaturing agent, unlike the gap in low avidity Abs [1]. As such, hydrophobic and ionic bonds stabilizing the complexes remain unbroken [31–33]. Since merozoite antigens have multiple epitopes, each of which induces Ab with different binding-strengths (affinity), AI represent the overall percentage of Ab with sufficient complementarity to remain bound after exposure to a specific chaotrope. Accordingly, the term “high avidity antibodies” is defined by the concentration of chaotrope used. For example, in Fig. 1a, plasma from Individual #3 would be described as having 82% and 75% high avidity Ab to AMA1 if 4 M GdHCl and 8 M were used, but only 8% high avidity to AMA1 in assays using 3 M NH_4_SCN. Likewise, in Fig. 2a, Individual #3 would be reported as having 95%, 30% and 8% high avidity Ab to AMA1 if 1 M, 2 M, or 3 M NH_4_SCN were used. Since “High Avidity Ab” is defined by the concentration of chaotrope, it is difficult to compare results between studies and interpret results when different protocols are used.

Today, bead-based MIA are commonly used to measure Ab levels to malarial antigens [22–26]. The MIA format has many advantages, including the requirement for small amounts of antigen, speed, assaying > 100 replicates (beads) instead of only a few wells, and being internally controlled since all antigens and reagents are present within the same well (e.g., if Ab are not detected for one antigen, but are to others, then absence of Ab is not due to a technical error). For these reasons, having an avidity MIA using the exclusion method would allow researchers to quickly obtain quantitative data about Ab avidity. Based on the initial results, however, it was not clear if an avidity MIA using the 5 antigens and a single chaotrope was feasible.

Follow-up experiments sought to further characterize and refine the bead-based MIA using different concentrations of chaotropes (Fig. 2 and Additional file 3: Fig. S3) and different incubation periods (Fig. 3). Overall, 2 M NH_4_SCN provided the widest range of AI for the 5 antigens. Since using a concentration of chaotrope that is in the center of the exclusion curve is desirable, 2 M NH_4_SCN was compared with the actual molar amount of NH_4_SCN needed to release 50% of bound Ab to each of the 5 antigens (Table 1). Using plasma from 40 adults living in a highly endemic malaria region, mean molar concentrations of NH_4_SCN to release 50% of bound Ab ranged from 1.7 to 2.3 M, with an average of 2.1 ± 0.32 M for the 5 antigens (Table 1). Thus, 2 M NH_4_SCN proved to be in the middle of the exclusion curves. Incubation with antigen-Ab complexes with 2 M NH_4_SCN for 15 or 30 min didn’t alter AIs significantly (Fig. 3), but the longer incubation period proved to be practical when running multiple plates. Using the avidity MIA, over 100 samples can be screened against the 5 or more antigens in a single afternoon with good reproducibility (Fig. 4). Since the assay provides information on both the amount (MFI) and the proportion of high avidity Ab (AI), data on both Ab quantity and quality is obtained in a single experiment. Without further experimentation, it is unclear if the protocol can be used for other malarial antigens, but results from this study provide a starting point for development of future avidity assays for other antigens.

In repeat experiments, AI values were more consistent than MFI (Fig. 4). In other words, AIs are technically more error-proof than raw MFIs. Technical and instrumental differences, for example due to pipetting errors or mis-calibration, result in variation of MFI when the same sample is used in multiple assays. However, the AI values are less affected since they measure the proportion of high binding antibodies. The wide dynamic range of MIA (e.g., 500 MFI to 25,000 MFI in this study) helps explain the very strong association between AI and the coefficient of variation (r = 0.935) (Fig. 4). Thus, these data support the feasibility of a simple, useful, repeatable avidity MIA for merozoite antigens.

Initially, it was hoped that by comparing the most commonly-used protocols in the same experiment, one might gain insight to help make comparisons and interpret data from previous studies. A conscientious search of the literature revealed that the 4 studies using 4 M GdHCl focused on merozoite antigens, including AMA1 [5, 6, 9, 11], MSP1 [5, 6, 9, 11], MSP2 [9], MSP3 [5]. Whereas, the 5 studies that used 8 M urea measured avidity to a schizont extract [2], *Plasmodium vivax* MSP1 and *P. vivax* Duffy Binding Protein [14, 17], and Ab from individuals vaccinated with AMA1 and Pf25 [15, 16]. In contrast, the 6 studies using SCN evaluated other malarial antigens including: a schizont-extract and 1 M NH_4_SCN [3], EBA-175 using 2.4 M NaSCN [8], the RTS/S vaccine and 1 M NH_4_SCN [12, 13], and VAR2CSA with 3 M NH_4_SCN [18, 19]. Thus, it does not appear that any of these prior studies are similar enough to be directly compared with each other. Accordingly, comparisons can be made within the same study among different cohorts or treatment groups, but direct comparison between studies remains unfeasible. Variation in methodologies and antigens used in previous studies may explain why an inconsistent picture of changes in Ab avidity with age and the role of Ab avidity in protection from malaria exist.

Results from this study provide hints about maturation of the Ab response in individuals living in malaria endemic areas. First, individuals may have high AI to one antigen but low AI to another antigen. For example, in Fig. 2, using 2 M NH_4_SCN, Individual #1 AI of 90 to MSP1-42, 85 to EBA-175, 25 to AMA1, but only 8% to MSP3. This result indicates that affinity maturation does not occur at the same rate (or reach the same level), for all antigens. Second, at the population level, Ab avidity tends to be greater for some antigens than others. For example, in Fig. 1, AI to MSP1-42 were high (> 90 with 3 M NH_4_SCN), but low AI to MSP2 and MSP3 (AI < 15 for 3 M NH_4_SCN). Thus, some antigens appear to induce affinity maturation better than others. Third, the amount of Ab was lower in infants than adults for the 5 merozoite antigens, requiring the use of 1:100 and 1:1000 dilutions of infant and adult plasma, respectively, for MFI to fall on the linear part of the binding curve. Interestingly, the amount of NH_4_SCN needed to release 50% of bound Ab was quite similar for Ab-positive infants and adults (Table 1), although lower amounts of chaotrope were required for infants for AMA1 and EBA-175, slightly lower for MSP1-42, but similar for MSP2 and MSP3 (Table 1). Studies in high transmission areas have reported little or no increase in Ab avidity with age for EBA-175, MSP1, MSP2, and MSP3 [4–9]. Plasma used in this study was from infants and adults living in a rural village with perennial transmission who received an estimated 257 infectious mosquito bites/person/year [29, 30]. Clearly, it would be interesting to investigate affinity maturation with age in this high transmission setting to determine if and when affinity maturation occurs. Overall, the use of 2 M NH_4_SCN in future studies evaluating maturation of immunity from infancy to adulthood seems appropriate; whereas, investigators studying acquisition of immunity in young children might consider using 1.5 M NH_4_SCN as the lower concentration will give a wider range of AI values.

## Conclusions

A multiplex bead-based avidity immunoassay is feasible for the merozoite antigens AMA1, EBA-175, MSP1-42, MSP2 and MSP3 that employs 2 M of NH_4_SCN. Further studies are needed to determine if 2 M NH_4_SCN is the best concentration of chaotrope for other malarial antigens. The assay is simple, measures avidity near the middle of the dynamic range, and AI are similar in repeated experiments. The assay provides a simple method to quickly obtain information about Ab quantity and quality in the acquisition of immunity to malaria in endemic populations.

## Supplementary information

**Additional file 1: Figure S1.** The Linear Range of the Ab Binding Curve. In a multiplex avidity MIA, it is important to establish the linear region of the Ab binding curve, where the amount of Ab is directly related to MFI. Simple linear regression was used to calculate r values.

**Additional file 2: Figure S2.** Similar Avidity Indexes (AI) are Obtained using Different Dilutions of Plasma. Since MFI is a measure of the amount of Ab; whereas, AI represent the strength of Ab binding to the antigen, AI should be independent on MFI. That is, AI should not change significantly when different dilutions of the same plasma sample are used in the assay, as long as the MFI are on the linear part of the Ab binding curve. In the avidity MIA reported in this study, MFI and AI were independent. Data above are provided to illustrate this point. Method: The same plasma samples used in Additional file 1: Fig. S1 that had different amounts of Ab (e.g., high > 20,000 MFI to lowest 1000 to 5000 MFI) for different antigens were selected. When the samples were diluted 1:100; 1:500, 1:1000 and 1:5000 and used in the avidity assay (Ab-Antigen-bead complexes were treated with 1 M NH4SCN for 30 min). Results: Results show that similar AI for were obtained using different dilutions of plasma. Thus, in the avidity multiplex assay described, MFI and AI were independent.

**Additional file 3: Figure S3.** Influence of Different Concentrations of Chaotropes on the Avidity Index (AI: Percentage of Antibodies that remained bound). Method: In this experiment, 50 µl of the diluted positive plasma control (PC) and 50 µl of the Ag coupled beads were incubated for 60 min; washed; beads were resuspended in 100 µl of the concentration of chaotrope shown for 30 min; washed; incubated with 100 µl of PE-anti-human IgG for 60 min; washed; and examined using a MicroChip 100.

## Data Availability

Essentially all data used and/or analysed in the current study are included in this publication. The data sets for individual experiments reported in this publication are available from the corresponding author on reasonable request.

## Abbreviations

Ab: Antibody/antibodies
AI/AIs: Avidity index/avidity indexes
AMA1: Apical merozoite antigen 1
CoV: Coefficient of variation
EBA-175: Erythrocyte binding antigen-175
GdHCL: Guanidine HCl
IRB: Institutional Review Board
MFI: Median fluorescence intensity
MIA: Bead-based, multiplex immunoassay
MSP: Merozoite surface protein
NC: Negative control
NH_4_SCN: Ammonium thiocyanate
SCN: Thiocyanate
SD: Standard deviation
PC: Positive control
PC#2: Second (alternate) positive control
r value: Coefficient of correlation using simple linear regression
