# Supplementary figures and images for: Measuring antibody avidity to Plasmodium falciparum merozoite antigens using a multiplex immunoassay approach
Source: Malar J. 2020 May 1;19:171. doi: 10.1186/s12936-020-03243-3 (PMC7195780; doi:10.1186/s12936-020-03243-3)

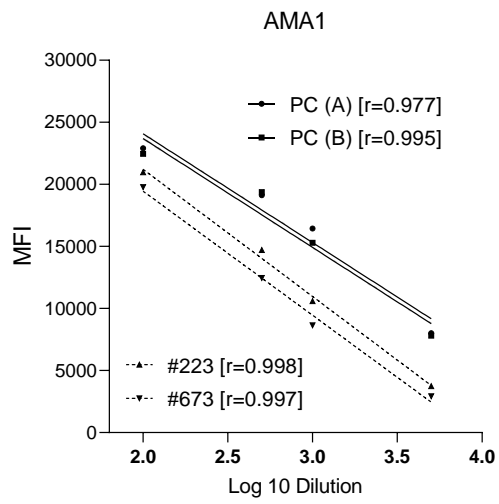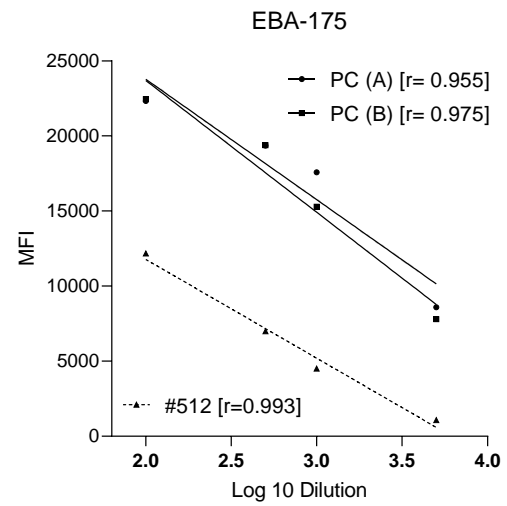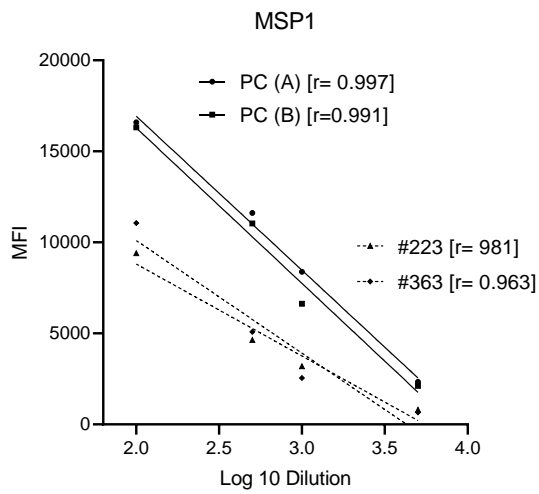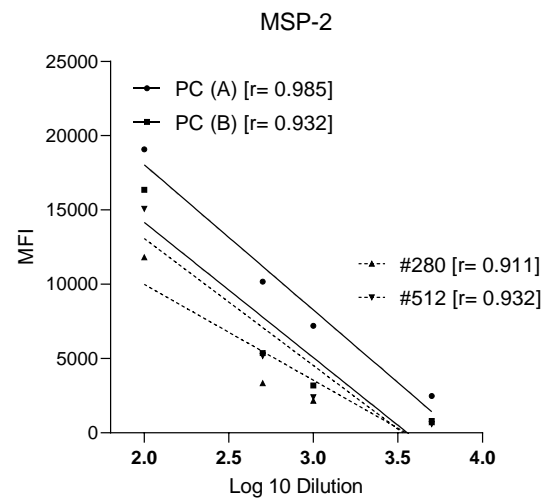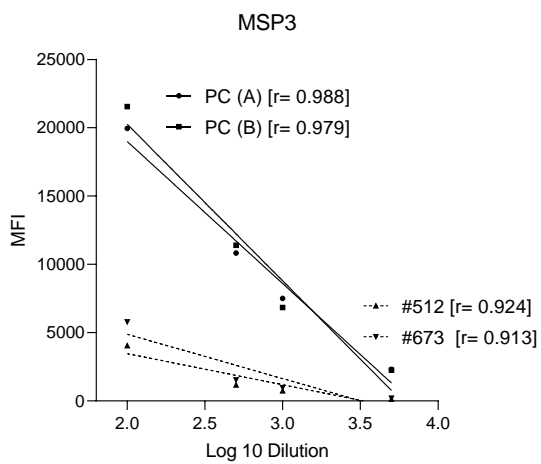

Supplement: Supplementary file 1 — Additional file 1: Figure S1. The Linear Range of the Ab Binding Curve. In a multiplex avidity MIA, it is important to establish the linear region of the Ab binding curve, where the amount of Ab is directly related to MFI. Simple linear regression was used to calculate r values. [file 12936_2020_3243_MOESM1_ESM.pdf]

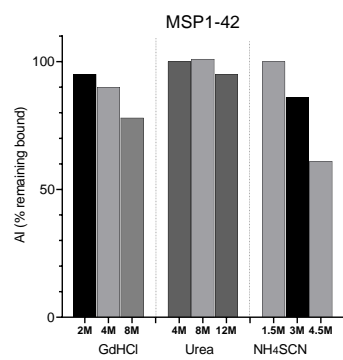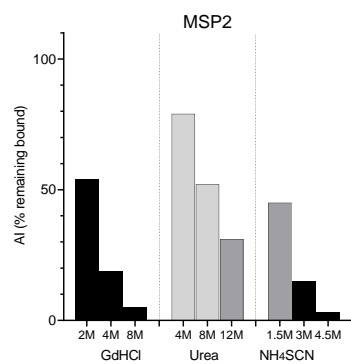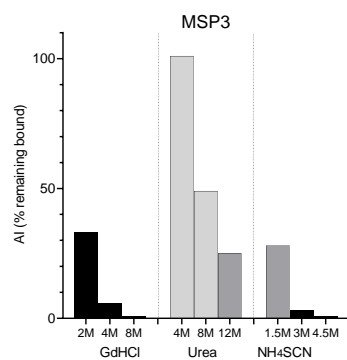

Supplement: Supplementary file 3 — Additional file 3: Figure S3. Influence of Different Concentrations of Chaotropes on the Avidity Index (AI: Percentage of Antibodies that remained bound). Method: In this experiment, 50 µl of the diluted positive plasma control (PC) and 50 µl of the Ag coupled beads were incubated for 60 min; washed; beads were resuspended in 100 µl of the concentration of chaotrope shown for 30 min; washed; incubated with 100 µl of PE-anti-human IgG for 60 min; washed; and examined using a MicroChip 100. [file 12936_2020_3243_MOESM3_ESM.pdf]
