# Supplementary material for: Measuring antibody avidity to Plasmodium falciparum merozoite antigens using a multiplex immunoassay approach
Source: Malar J. 2020 May 1;19:171. doi: 10.1186/s12936-020-03243-3 (PMC7195780; doi:10.1186/s12936-020-03243-3)
Supplement: Supplementary file 2 — Additional file 2: Figure S2. Similar Avidity Indexes (AI) are Obtained using Different Dilutions of Plasma. Since MFI is a measure of the amount of Ab; whereas, AI represent the strength of Ab binding to the antigen, AI should be independent on MFI. That is, AI should not change significantly when different dilutions of the same plasma sample are used in the assay, as long as the MFI are on the linear part of the Ab binding curve. In the avidity MIA reported in this study, MFI and AI were independent. Data above are provided to illustrate this point. Method: The same plasma samples used in Additional file 1: Fig. S1 that had different amounts of Ab (e.g., high > 20,000 MFI to lowest 1000 to 5000 MFI) for different antigens were selected. When the samples were diluted 1:100; 1:500, 1:1000 and 1:5000 and used in the avidity assay (Ab-Antigen-bead complexes were treated with 1 M NH4SCN for 30 min). Results: Results show that similar AI for were obtained using different dilutions of plasma. Thus, in the avidity multiplex assay described, MFI and AI were independent. [file 12936_2020_3243_MOESM2_ESM.pdf]

Figure S2:

Similar Avidity Indexes (AI) are Obtained using Different Dilutions of Plasma

>20,000 MFI with dilution				
	Dilution	no salt	1M salt	AI
AMA1	1:100	23623	22283	94%
	1:500	18769	17652	94%
	1:1000	15428	13729	89%
			Average	**92%**
			SD	**3%**
EBA-175	1:100	22457	22650	101%
	1:500	19382	18519	96%
	1:1000	15286	14233	93%
			Average	**97%**
			SD	**4%**

**15,000 - 20,000 MFI with Dilution**				
	Dilution	no salt	1M salt	AI
MSP1	1:100	16314	15464	95%
	1:500	11037	9008	82%
	1:1000	6625	5736	87%
			Average	**88%**
			SD	**5%**
MSP3	1:100	17294	15894	92%
	1:500	8049	6984	87%
	1:1000	5332	4506	85%
			Average	**88%**
			SD	**4%**
AMA1	1:100	1:100	1:100	85%
	1:500	1:500	1:500	75%
	1:1000	4921	3840	78%
			Average	**79%**
			SD	**5%**

**10,000 - 15,000 MFI with Dilution**

	Dilution	no salt	1M salt	AI
EBA-175	1:100	12169	10750	88%
	1:500	6990	6296	90%
	1:1000	4503	4066	90%
			Average	**90%**
			SD	**1%**
MSP-1	1:100	11064	1260	11%
	1:500	5081	420	8%
	1:1000	2549	215	8%
			Average	**9%**
			SD	**2%**
MSP2	1:100	12042	4343	36%
	1:500	5611	2008	36%
	1:1000	4660	1833	39%
			Average	**37%**
			SD	**2%**

**5,000 - 10,000 MFI with Dilution**				
	Dilution	no salt	1M salt	AI
MSP1	1:100	8891	7550	85%
	1:500	5237	3993	76%
	1:1000	3796	2938	77%
			Average	**80%**
			SD	**5%**
MSP3	1:100	5791	659	11%
	1:500	1533	117	8%
	1:1000	954	95	10%
			Average	**10%**
			SD	**2%**

**1,000 - 5,000 MFI with Dilution**				
	Dilution	no salt	1M salt	AI
MSP3	1:100	4054	790	19%
	1:500	1172	172	15%
	1:1000	747	102	14%
			Average	**16%**
			SD	**3%**

EBA-175	1:100	2778	1738	63%
	1:500	1216	787	65%
	1:1000	689	369	54%
			Average	**60%**
			SD	**6%**
